# Age-Related Molecular Polymorphism of the Heterodimeric Proteoglycan Bisdermican

**DOI:** 10.1100/tsw.2004.204

**Published:** 2004-11-30

**Authors:** Martin Götte, David D. Sofeu Feugaing, Hans Kresse

**Affiliations:** Departments of Obstetrics and Gynecology, Münster University Hospital, Münster, Germany; ^2^ Departments of Physiological Chemistry and Pathobiochemistry, Münster University Hospital, Münster, Germany

**Keywords:** glycosaminoglycan, dermatan sulfate, developmental regulation, aging, glycosylation

## Abstract

Bisdermican (PG760) is a large, heterodimeric, dermatan sulfate proteoglycan found in selected basement membranes, smooth muscle cell layers, and different extracellular matrices. Age-dependent and developmentally regulated alterations in glycosaminoglycan structure and quantity have been shown to be functionally relevant for a number of physiological and pathological processes. Bisdermican was purified from human skin fibroblast cultures of different age and confluency. Following β-elimination, glycosaminoglycan chains were analyzed by Sephacryl-S-300 chromatography. Glycosaminoglycan chains of Bisdermican from infantile fibroblasts had a molecular weight of 19 kDa, whereas the glycosaminoglycan chain of the large Bisdermican subunit purified from confluent fetal fibroblast secretions was slightly larger (M_r_ = 24 kDa). Bisdermican derived from subconfluent cultures of fetal fibroblasts displayed the largest glycosaminoglycan chains with a molecular weight of 31.5 kDa for the large subunit, and a molecular weight of 22 kDa for the small subunit. Thus, Bisdermican displays a molecular polymorphism that is related to its chronological age and proliferative state.

## INTRODUCTION

Proteoglycans are a class of glycoproteins of the extracellular matrix and cell surfaces that are characterized by the presence of one or several glycosaminoglycan chains, which are covalently attached to a core protein. Glycosaminoglycans are long, unbranched, anionic polysaccharides containing repetitive disaccharide units. These disaccharide units contain two modified carbohydrates. With the exception of the galactose-containing keratan sulfate, N-acetylgalactosamine or N-acetylglucosamine is linked to a uronic acid such as glucuronic acid or iduronic acid[1,2,3]. Depending on the disaccharide composition and the degree of epimerization and sulfation, dermatan/chondroitin sulfate, heparan sulfate/heparin, keratan sulfate, and hyaluronic acid can be distinguished. Proteoglycans play major roles in a variety of physiological processes and are no longer only considered structural components. For example, proteoglycans have been implicated in the regulation of cell adhesion, migration, differentiation, and signaling in inflammation, development, wound repair, and tumorigenesis[1,2,3,4,5,6]. While some proteoglycan functions are mediated by the core protein, a major part is mediated by the glycosaminoglycans, which can form ligand binding sites defined by the degree of epimerization and sulfation, and the organization into domain structures[2].

Changes in the content and composition of glycosaminoglycans have been observed in many processes, including embryonic development, pathogenesis, and aging. Brain proteoglycans and their glycosaminoglycan chains display a highly regulated spatiotemporal expression during development and maturation of the nervous system[7,8]. During development of the embryonic chicken brain, the total amounts of both chondroitin sulfate chains and the activity of glycosaminoglycan-modifying sulfotransferases were highest during the early embryonic stages and decreased sharply as the development reached completion[9]. A differential increase in glycosaminoglycan synthesis has also been observed during gastric glandular morphogenesis in the chick embryo, where an increase in radioactive sulfate incorporation was noted between days 13 and 18 of development[10]. The functional consequences of a loss of glycosaminoglycans for developmental processes have been demonstrated in a number of experiments. An inhibition of heparan sulfation results in profound effects on spinal neurulation[11], whereas an enzymatic removal of chondroitin sulfate abolishes the age-related reorganization of retinal axons in mouse embryos[12]. Furthermore, distinct alterations in sulfation patterns and total chain length of heparan sulfate during maturation of the embryonic brain correlate with the ability of heparan sulfate to potentiate the activity of fibroblast growth factors[13].

Several changes in glycosaminoglycan composition and quantity have been described in the context of aging. In human articular cartilage, the structure of proteoglycans changes considerably between the fetus and the mature adult, leading to an increase in protein relative to glycosaminoglycan and a decrease in proteoglycan size (i.e., a decrease in glycosaminoglycans)[14]. Age-related decreases in glycosaminoglycans have also been noted in the human vocal ligament tendon[15], the human lamina cribrosa[16], and the lenticular and humoral side of human lens capsules in comparison with young lens capsules[17]. Furthermore, age-related changes in glycosaminoglycan content are seen in the skeletal muscle of normal and dystrophic mice aged from 3–18 weeks. In normal muscle, the amount of glycosaminoglycan decreased slightly in the period from 3–10 weeks, and changes in the relative amounts of different glycosaminoglycan types were observed. Interestingly, the incorporation of [$^{35}$S]sulfate into the dystrophic muscle, which had a higher glycosaminoglycan content, was only about 60% of normal muscle[18]. However, one has to keep in mind that the expression of a number of proteoglycan core proteins is also changed in dystrophic muscle[19].

In this study, we describe an age-related molecular polymorphism of the heterodimeric basement membrane proteoglycan Bisdermican (PG-760). Bisdermican was initially isolated by Breuer et al.[20] as a major proteoglycan species of fetal human skin fibroblasts, but it is also found in endothelial cells and keratinocytes[21]. It is composed of two core proteins of 460 and 300 kDa, respectively, which are probably encoded by different genes. In fetal fibroblasts, the core proteins are substituted with one or very few dermatan sulfate chains of similar chemical composition[20]. Immunogold labeling of Bisdermican demonstrated its localization in fibrils on the cell surface as well as in fibrillar extensions from the cell body. It could also be demonstrated that Bisdermican colocalizes with fibronectin, laminin, perlecan, and type IV collagen, suggesting an association with basement membranes[21]. This association could be confirmed by immunostaining of different mouse tissues, where Bisdermican was found in selected basement membranes[22]. The presence of Bisdermican was demonstrated in the cornea epithelium including the basement membrane, stroma, and Descemet's membrane, in the skin, the mucosa of the small intestine, EHS tumor matrix and cells, and the smooth muscle layers of uterus, small intestine, and blood vessels. No staining was seen in capillaries, striated muscles, and liver parenchyma including the central vein. In a previous study[20], we had observed an increased electrophoretic mobility of both subunits of Bisdermican isolated from infantile fibroblasts compared to fetal fibroblasts. Moreover, differences in the electrophoretic behavior of subconfluent and confluent cultures were noted, respectively. Here, we confirm and extend our previous finding in molecular detail. Using Sephacryl-S-300 gel filtration chromatography, we demonstrate an increase in glycosaminoglycan chain length of the large subunit of Bisdermican isolated from fetal fibroblasts (24 kDa) relative to infantile fibroblasts (19 kDa). In addition, we show that the glycosaminoglycan chain length of Bisdermican of both subunits isolated from subconfluent fetal fibroblast cultures (31.5 kDa/22 kDa) is increased relative to Bisdermican of confluent fetal and infantile fibroblasts. Thus, Bisdermican displays a molecular polymorphism that is related to its chronological age and proliferative state.

## MATERIALS AND METHODS

### Cell Culture

Human skin fibroblasts from a 6-week-old infantile donor and from fetal skin (obtained by courtesy of Drs. W. Holzgreve and H. Lücke, Department Gynecology, Münster University Hospital) were maintained in modified Eagle's medium essential medium containing 10% fetal calf serum, nonessential amino acids, and antibiotics as described[23]. Cells were cultured at 37°C in the presence of 5% $CO_2$. This work was conducted in conformity with the principles embodied in the Declaration of Helsinki.

### Purification of Metabolically [$^{35}$S]Sulfate-Labeled Bisdermican

Bisdermican was purified as described previously[20]. Cells of the fifth to sixth passage were plated 1–2 days prior to metabolic labeling at a density that resulted in confluent or subconfluent cultures, respectively, at the time of labeling. Cells were incubated for 16 h with 1.5 MBq/ml of [$^{35}$S]sulfate (carrier-free, Amersham-Buchler, Braunschweig, Germany) in a medium free of streptomycin sesquisulfate. Subsequently, the conditioned medium was supplemented with protease inhibitors and made 70% saturated with ammonium sulfate. The precipitate was dissolved in 700 µl/75-cm² culture flask of 100 m*M* Tris/HCl buffer, pH 7.4, containing 1 *M* HCl, 0.5% sodium desoxycholate, 0.5% Triton-X-100, 0.2% SDS, and protease inhibitors, and centrifuged for 15 min at 12,000 × *g*. For immunoprecipitation of Bisdermican, the supernatant was sequentially treated with protein A-Sepharose (Sigma, Taufkirchen, Germany) coated with IgG from control and antiserum, as described[20].

### Agarose Gel Electrophoresis and β-Elimination of Glycosaminoglycan Chains

[$^{35}$S]Sulfate-labeled Bisdermican was obtained by immunoprecipitation. Samples were incubated in 24 m*M* Tris, pH 8.3, 192 m*M* glycine, 0.1% SDS, and 0.01% bromophenol blue for 30 min at 37°C. Subsequently, 20 m*M* dithiothreitol were added for 30 min to disassemble the disulfide-linked heterodimeric proteoglycan. The sample was subjected to agarose-gel electrophoresis at 7 V/cm in a 1.5% agarose (Sigma Taufkirchen, Germany) gel containing 24 m*M* Tris, pH 8.3, 192 m*M* glycine, and 0.1% SDS. As molecular weight markers, the laminin A- and B1-chains and nidogen (kindly provided by Dr. A. Ries, Max-Planck-Institute of Biochemistry, Martinsried, Germany) were used. After the migration distance of bromophenol blue had reached 10 cm, Bisdermican was electrophoretically transferred onto a DEAE-membrane (NA 45, Schleicher&Schuell, Germany) preabsorbed with 3% bovine serum albumin in 50 m*M* Tris/HCl pH 7.5, 150 m*M* NaCl. Transfer was performed at 7 V/cm for 45 min. After washing with water, the membrane was dried at 60°C. The two subunits of Bisdermican were localized by autoradiography using Kodak X-OMAT film, and the respective bands cut out of the membrane. Intact glycosaminoglycans were released from the core proteins by alkaline borohydride treatment (β-elimination) (0.1 *M* NaOH. 1 *M* $NaBH_4$, 20 h at 37°C). The reaction was stopped by the addition of 50% acetic acid, resulting in pH 5, and the glycosaminoglycan chains were subjected to gel filtration chromatography.

## Sephacryl-S-300 Gel Filtration Chromatography

Gel filtration of [$^{35}$S]sulfate-labeled glycosaminoglycans was performed on a calibrated Sephacryl-S-300 (Pharmacia LKB, Germany) column (1 × 90 cm), in 50 m*M* sodium acetate, pH 5.8, 4 *M* guanidinium chloride, and 0.1% Triton X-100. $V_0$ was determined using dextran blue, followed by photometric detection. $V_t$ was determined using [$^{35}$S]sulfate, followed by liquid scintillation counting as described[23]. Reference glycosaminoglycans[20] of 37 kDa ($k_{av}$ = 0.208), 19 kDa ($k_{av}$ = 0.414), and 12.4 kDa ($k_{av}$ = 0.522) were used to determine the molecular weight of Bisdermican glycosaminoglycan chains.

## RESULTS

In a previous study[20], we had observed an increased electrophoretic mobility of both subunits of Bisdermican isolated from infantile fibroblasts compared to fetal fibroblasts. In addition, differences in the electrophoretic behavior of subconfluent and confluent cultures were noted, respectively. Moreover, the proteoglycan bands exhibited a more discrete appearance, which would be in accordance with Bisdermican carrying fewer and/or shorter glycosaminoglycan chains. In order to analyze this observation in molecular detail, we purified the glycosaminoglycan chains of both subunits of Bisdermican from the secretions of confluent and subconfluent cultures of fetal fibroblasts[20] and a newly established culture of infantile fibroblasts from a 6-week-old healthy donor. Agarose gel electrophoresis of immunoprecipitated, [$^{35}$S]sulfate-labeled Bisdermican from the cultures of these infantile fibroblasts was followed by electrotransfer to a DEAE-membrane and autoradiography (Fig. 1). As was previously observed for infantile fibroblast of donors aged 4 and 9 years[20], the bands display an appearance that is quite discrete for a proteoglycan, showing only moderate smearing. We next isolated the glycosaminoglycan chains of both subunits of Bisdemican from this source by β-elimination and determined the molecular weight by gel filtration chromatography on a Sephacryl-S-300 column (Fig. 2). Both subunits carried glycosaminoglycan chains of 19 kDa (Table 1). We next purified the glycosaminoglycan chains of Bisdermican obtained from confluent cultures of fetal fibroblasts[20] in an analogous way (Fig. 3). In contrast to the glycosaminoglycan chains of infantile fibroblasts, the glycosaminoglycan chain of the large subunit of Bisdermican was larger (24 kDa) than the glycosaminoglycan chain of the smaller subunit, which displayed the same molecular weight as the glycosaminoglycan of the small subunit of Bisdermican from infantile fibroblasts (19 kDa, Table l). Since we had previously noted differences in the electrophoretic mobility of Bisdermican subunits purified from subconfluent and confluent cultures of fetal fibroblasts[20](Götte M. and Kresse H., unpublished), we analyzed the glycosaminoglycan chains of Bisdermican purified from the conditioned media of subconfluent fetal fibroblasts. Sephacryl-S-300 gel filtration chromatography revealed an increase in the molecular weight of glycosaminoglycans from both Bisdermican subunits compared to Bisdermican purified from confluent cultures of fetal fibroblasts (Fig. 4). While the glycosaminoglycan chain of the small subunit was only slightly larger than the glycosaminoglycan chain of the small subunit purified from confluent cultures of both fetal and infantile fibroblasts (22 kDa vs. 19 kDa, Table 1), a considerable increase in the size of the glycosaminoglycan of the large subunit was observed (31.5 kDa vs. 24 kDa or 19 kDa, respectively, Table 1). Thus, Bisdermican displays a molecular polymorphism, which is due to a differential glycosylation of its core protein(s). These differences correlate both with the chronological age and proliferative phase of the cells synthesizing this heterodimeric basement membrane proteoglycan.

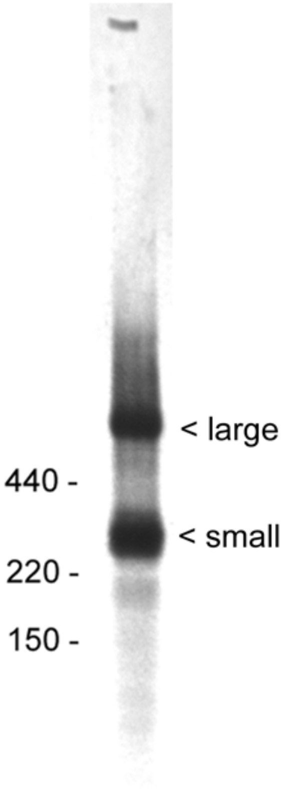

**FIGURE 1**. Fluorogram of [$^{35}$S]sulfate-labeled Bisdermican after SDS-agarose gel electrophoresis. Bisdermican was purified from the culture medium of confluent fifth passage infantile fibroblasts by immunoprecipitation after a labeling period of 16 h in the presence of 1.5 MBq [$^{35}$S]sulfate/ml. The sample was treated with dithiothreitol (DTT) and iodoacetamide. The numbers indicate $10^{-3} \times M_r$ of the reference proteins laminin A and B1 chain and nidogen. Arrows indicate the large and small subunits of Bisdermican, respectively.

## DISCUSSION

In this study, we confirm and extend our previous observation of an altered electrophoretic mobility of the subunits of Bisdermican derived from human fibroblasts of different chronological age and proliferative state. Using Sephacryl-S-300 gel filtration chromatography, we could demonstrate that Bisdermican subunits from infantile donor fibroblasts show a relatively focused migration behavior in agarose gel electrophoresis, which would be conform with less glycanation compared to fetal Bisdermican. Bisdermican from infantile fibroblasts carries glycosaminoglycan chains of an identical molecular weight of 19 kDa (Table 1). In contrast, confluent fetal fibroblast Bisdermican carried a slightly larger glycosaminoglycan chain of 24 kDa on its large subunit, which is in accordance with our previous data[20]. This difference was even more pronounced in the case of subconfluent fetal fibroblasts, demonstrating a clear molecular polymorphism of fibroblast Bisdermican. A good example for another proteoglycan that displays a molecular polymorphism is syndecan-1, a cell surface proteoglycan that plays major roles in the regulation of inflammatory events, and that is differentially expressed during epithelial-mesenchymal transformation[1,4,5,6]. Not only has a hypoglycanated murine syndecan-1 splice variant been described[24], the size of the heparan sulfate chains of syndecan-1 can also vary between 20 kDa and 70 kDa, depending on the cell type and tissue where syndecan-1 is expressed. This difference

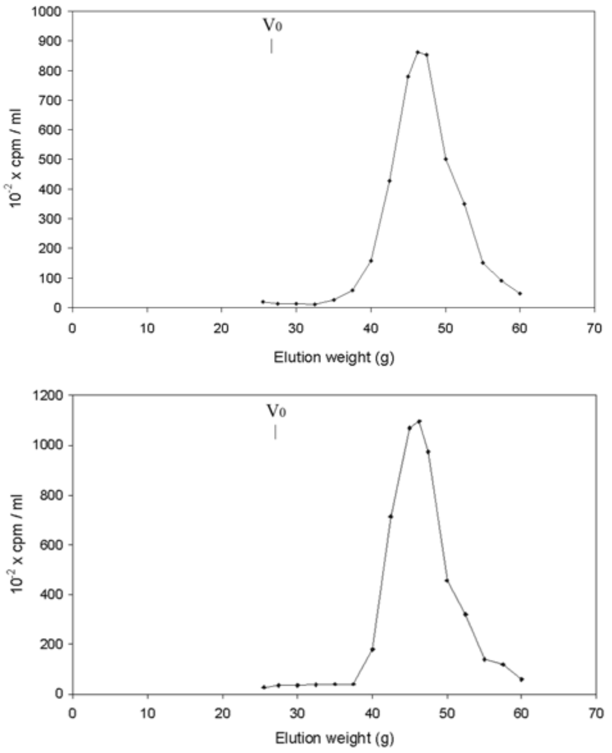

**FIGURE 2**. Chromatography of [$^{35}$S]sulfate-labeled glycosaminoglycans derived from Bisdermican isolated from infantile fibroblast secretions on a Sephacryl-S-300 column. Glycosaminoglycans were obtained from the secretions of confluent infantile fibroblast cultures after a labeling period of 16 h in the presence of 1.5 MBq [$^{35}$S]sulfate/ml. Glycosaminoglycans that were liberated from the core proteins of the large subunit (upper panel) or small subunit (lower panel) of Bisdermican were sequentially chromatographed on a Sephacryl-S-300 column (total elution weight 73 g). See Table 1 for molecular weight of the glycosaminoglycan chains.

**TABLE 1**
**Molecular Weight of Bisdermican Glycosaminoglycan Chains**

| | Large Subunit | Small Subunit |
|---|---|---|
| Infantile fibroblasts | 19 kDa | 19 kDa |
| Fetal fibroblasts | 24 kDa | 19 kDa |
| Fetal fibroblasts (subconfluent) | 31.5 kDa | 22 kDa |

[$^{35}$S]Sulfate-labeled glycosaminoglycans were isolated from the secretions of confluent cultures of infantile and fetal fibroblasts, and subconfluent fetal fibroblasts, respectively. The molecular weight of the glycosaminoglycan chains was calculated from the Sephacryl-S-300 chromatography peaks shown in Figs. 2–4 using the elution maxima of reference glycosaminoglycans of 37 kDa, 19 kDa, and 12.4 kDa as described[20].

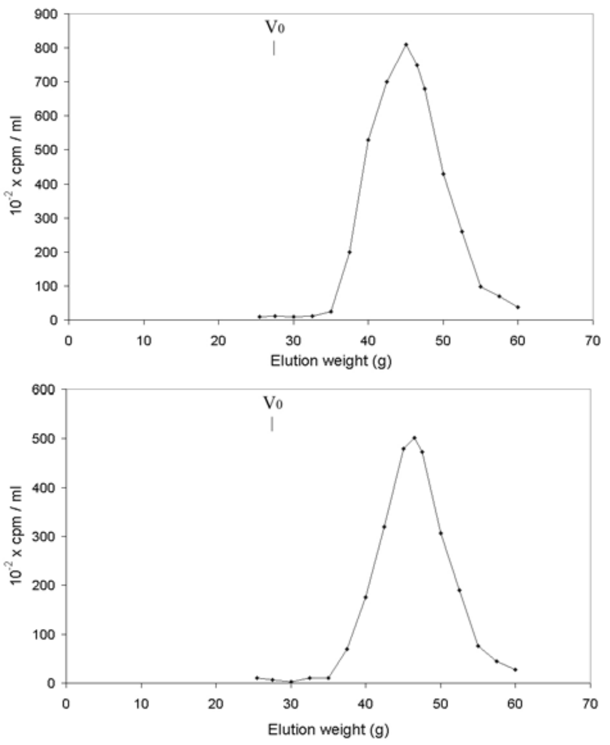

**FIGURE 3**. Chromatography of [$^{35}$S]sulfate-labeled glycosaminoglycans derived from Bisdermican isolated from confluent fetal fibroblast secretions on a Sephacryl-S-300 column. Glycosaminoglycans were obtained from the secretions of confluent fetal fibroblast cultures after a labeling period of 16 h in the presence of 1.5 MBq [$^{35}$S]sulfate/ml. Glycosaminoglycans that were liberated from the core proteins of the large subunit (upper panel) or small subunit (lower panel) of Bisdermican were sequentially chromatographed on a Sephacryl-S-300 column (total elution weight 73 g). See Table 1 for molecular weight of the glycosaminoglycan chains.

has functional consequences and does, for example, account for different binding affinities of syndecan-1 to collagen I or tenascin, respectively[1,25,26]. Bisdermican shows a codistribution with several basement membrane components[21] and it localizes to selected basement membranes[22]. One could envisage that the polymorphism of Bisdermican glycosaminoglycans could lead to alterations in the binding affinity of Bisdermican to basement membrane components during embryonic development. This would be a means of regulating basement membrane assembly during embryogenesis.

A more pronounced difference in glycosaminoglycan chain length between fibroblast groups was observed for the large subunit of Bisdermican. Since the subunits of Bisdermican are most likely encoded by two different genes[20], one could envisage that the small and large subunits contain either different glycosylation consensus sequences, or that they take different trafficking routes through the intracellular glycosylation machinery. Intracellular vesicular transport relies on highly conserved mechanisms and is tightly regulated[27,28]. Previous studies on the kinetics of Bisdermican biosynthesis had shown that glycosaminoglycan biosynthesis is a late event during the intracellular transport of the heterodimeric protein[20]. Therefore, it is more likely that the different core proteins become differentially substituted with glycosaminoglycans due to structural properties of the core proteins that mediate recognition by glycosylating enzymes, than by differential trafficking through the secretory pathway[2,27,29].

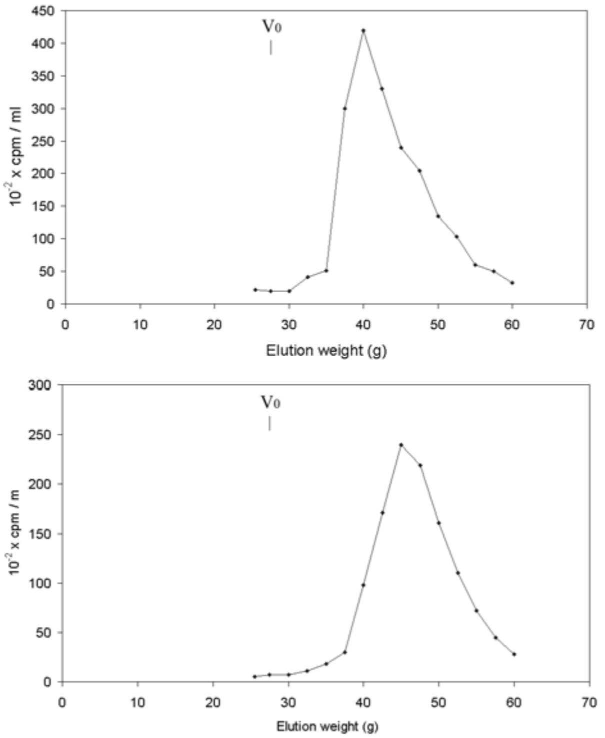

**FIGURE 4**. Chromatography of [$^{35}$S]sulfate-labeled glycosaminoglycans derived from Bisdermican isolated from subconfluent fetal fibroblast secretions on a Sephacryl-S-300 column. Glycosaminoglycans were obtained from the secretions of subconfluent fetal fibroblast cultures after a labeling period of 16 h in the presence of 1.5 MBq [$^{35}$S]sulfate/ml. Glycosaminoglycans that were liberated from the core proteins of the large subunit (upper panel) or small subunit (lower panel) of Bisdermican were sequentially chromatographed on a Sephacryl-S-300 column (total elution weight 73 g). See Table 1 for molecular weight of the glycosaminoglycan chains.

Subconfluent fetal fibroblast displayed the largest glycosaminoglycan chains of all Bisdermican samples investigated (Table 1). The phenotype of subconfluent fetal fibroblasts is of a migratory, proliferative nature. Therefore, it shows a closer resemblance to migrating cells during embryonic development than a more resting, confluent culture. This would be in accordance with the observation that fetal chondrocytes in alginate culture produce a more abundant cellular matrix that is richer in proteoglycans than the cellular matrix of young or adult cells[30]. The same concept of a migratory, proliferative nature of subconfluent vs. more resting, confluent cells would also apply to infantile fibroblasts, however, at present we can only speculate that the glycosaminoglycan chains of Bisdermican isolated from subconfluent cultures may be larger than Bisdermican glycosaminoglycan chains of confluent infantile fibroblasts.

In the study by Kamada et al.[30], it was also noted that the size of the glycosaminoglycan hyaluronic acid decreased markedly with age. In fact, a number of post-translational modifications of glycosaminoglycans, such as epimerization, N-sulfation, and O-sulfation have been demonstrated to change in human bone proteoglycans in an age-dependent manner[31]. Interestingly, osteoblasts from patients with osteogenesis imperfecta do not display signs of premature aging, but appear to be arrested in a fetal-like phenotypic state[31], demonstrating the importance of changes in glycosaminoglycan structure. Apart from changes in the binding affinity to different ligands, changes in the susceptibility to protease cleavage has been noted as one functional aspect of age-related modification of glycosaminoglycan patterns[32]. Given the important role of the basement membrane in preventing metastasis of tumors, in keeping the integrity of epithelial sheets, and in glomerular filtration[33,34,35], several potential functions could be assigned to the molecular polymorphism of the heterodimeric basement membrane proteoglycan Bisdermican. Our data are in accordance with a large number of studies showing age- and developmental stage-dependent changes in the glycosylation of proteoglycans. This study points out future directions of a functional characterization of Bisdermican, which will focus on the role of this proteoglycan during embryogenesis.

## ACKNOWLEDGMENTS

This project was financially supported by the Deutsche Forschungsgemeinschaft (SFB 310, project B2 and SFB 492, project A6). The authors would like to thank Sylvia Teich for assistance with cell culture, and Dr. Burkhard Breuer for discussions.
